# Influence of the Laser Cladding Parameters on the Morphology, Wear and Corrosion Resistance of WC-Co/NiCrBSi Composite Coatings

**DOI:** 10.3390/ma14195583

**Published:** 2021-09-26

**Authors:** Iosif Hulka, Ion D. Uțu, Diana Avram, Mircea L. Dan, Alexandru Pascu, Elena M. Stanciu, Ionuț C. Roată

**Affiliations:** 1 Research Institute for Renewable Energie, Politehnica University of Timişoara, G. Muzicescu 138, 300501 Timişoara, Romania; iosif.hulka@upt.ro; 2 Faculty of Mechanics, IMF Department, Politehnica University of Timişoara, Blvd. M. Viteazu 1, 300222 Timişoara, Romania; dragos.utu@upt.ro; 3 Faculty of Industrial Chemistry and Environmental Engineering, CAICAM Department, Politehnica University of Timişoara, Blvd. V. Pârvan, nr. 6, 300223 Timişoara, Romania; diana.avram@student.upt.ro (D.A.); mircea.dan@upt.ro (M.L.D.); 4 Materials Engineering and Welding Department, Transilvania University of Braşov, Eroilor Blvd., 29, 500036 Braşov, Romania; elena-manuela.stanciu@unitbv.ro (E.M.S.); ionut.roata@unitbv.ro (I.C.R.)

**Keywords:** WC-Co/NiCrBSi, laser cladding, wear resistance, corrosion, cermet

## Abstract

To enhance the sliding wear and corrosion behavior of steels with low carbon content, cermet composite coatings are usually deposited on their surface by various deposition processes. Laser cladding, compared to other deposition techniques such as electroplating, arc welding, and thermal spraying, has numerous advantages to produce such protective coatings. The paper presents the optimization of laser cladding deposition speed versus energy density in order to obtain WC-Co/NiCrBSi coatings with Ni-Al addition free of defects and reduced porosity deposited on low carbon steel substrate. The microstructure and chemical composition were investigated by SEM combined with EDX analysis while XRD was performed in order to examinate the phases within the coatings. In order to investigate the cladding speed influence on the coatings, hardness measurements, POD (pin on disk) wear tests and corrosion tests in 3.5% NaCl solution were carried out. The results showed that an optimal cladding speed has a crucial impact on the microstructure, composition, and hardness. It was found out that optimizing the cladding deposition speed proved to be effective in enhancing the sliding wear resistance and corrosion behavior by controlling the iron content within the coatings.

## 1. Introduction

Surface degradation due to wear and corrosion or their synergistic effect cost industrial economies hundreds of billions of dollars each year [1]. In order to reduce this enormous cost, there are several engineering solutions used to prevent wear and corrosion (e.g., carburizing, nitriding, deposition of protective coatings).

Modern techniques enable the production of different types of coatings from simple to complex ones using various composite materials. The most used techniques reported in the literature are thermal spraying, plasma spraying and laser cladding [2,3].

Laser cladding became a popular technology for obtain thick layers with improved mechanical properties, corrosion resistant and has the advantage of an easy control of deposition parameters, low dilution and a very good adherence to the substrate. Nowadays, by using the laser technology is possible to fabricate coated layers by preplaced method or by coaxial injection of the powder. Moreover, using the CAD design and robotics technology is possible to create new complex surfaces or precise recondition of worn components by using coaxial laser cladding technology. There is a wide range of powders and limitless possibility of alloys mixture by frequently using Ni, Co, or Fe [4,5,6] as base matrix with the addition of elements (Si, Ti, Cr, B, V) [4,7,8,9], carbides (TiC, WC, VC) [10,11] or ceramics (Al_2_O_3_, TiO_2_) [12,13]. All the above presented powders formerly designed for thermal spraying (e.g., high velocity oxygen spraying, flame spraying, plasma deposition) are studied nowadays and used for advanced laser cladding process. From the wide range of alloyed powders, considerable attention is attributed to NiCrBSi (Ni-based) and Stellite 6 (Co-based) powders which are relatively low-cost and can provide high-quality cladded layers. There are numerous research studies related to laser cladding of WC reinforcement in an either Ni [14,15,16,17,18,19] or in Co matrix [20,21,22,23], compared with coatings fabricated by a mixed Ni-Co matrix which are less investigated. The WC is well known for its high hardness and wear resistance and Co for the wetting capability and the capacity to dissolve and to form eutectic with WC (1275 °C/1350 °C). In their work, Fernández et al. [24] carry out a comprehensive study on the addition of WC in different percentage within a NiCrBSi matrix. They concluded that by using up to 30% WC the wear resistance was significantly improved. In a similar research Zhou et al. [25] used an induction laser cladding and 35% WC addition in order to obtain crack-free NiCrBSi composite coatings. Avoiding the complete dissolution of WC into the matrix was possible by hybrid processing induction–laser cladding which allows increased cladding speeds. Similar results, based on numerical and experimental study were reported by Farahmand [26].

Using a cobalt matrix, Paul et al. [21] obtained crack free and dense WC-Co cladded layers on steel substrate with no dissolution of hard carbides into the matrix by using a pulsed laser. A practical application of WC-Co hardfacing on B27 boron steel is presented by Bartkowski [22]. In his study the wear resistance of the cobalt-based coatings was successfully tested in real condition showing that laser cladding is well suited for application where hard coatings with high surface roughness are demanded.

Despite the fact that there are numerous advantages, the major drawback of the harfacing layers, especially of those reinforced with WC, is the internal stresses and the cracking susceptibility that occurs during solidifications. This issue can limit the application of the hard composite materials. In a recent study, Lee et al. [27] investigate the crack susceptibility and internal stress within a NiCr matrix with WC-Co addition. Their results showed that the WC-Co cermet is directly responsible for the formation of cracks and micro-pores during solidification especially at the boundary of the WC phase, which will lead to the formation of brittle fractures within the coating. Enhancing the properties and quality of hard composite coatings can be done by fine tuning of the cladding parameters or by selecting properly the feedstock powders. Preheating, optimizing the process parameters, adding an intermediate layer, using functionally graded layers with various hard particles percent [15,16,26,28,29] are several methods currently investigated to mitigate the internal stress and crack formation within the coatings.

Combining the toughness and corrosion resistance of NiCrBSi alloy with the high hardness and wear resistance of WC-Co hard phase is a good solution for obtaining protective coatings with improved mechanical behavior and a low cracking susceptibility. In a previous study Staicu et al. [30] investigated the laser cladding of NiCrBSi + WC-Co coatings on an AISI 5140 substrate. Good results were revealed but the characterization of the obtained coatings was limited to single tracks which are less susceptible to cracks formation.

The aim of this study is to further investigate the laser cladding of NiCrBSi feedstock powder with addition of Ni-Al as matrix for WC-Co hard phase. The interest for this composition is due to the fact that it was not properly studied. In the literature there is not much information about this particular composition deposited by laser cladding. In our study, single and partially overlapped tracks were fabricated by laser cladding using different deposition speeds. The obtained tracks and coatings were investigated by SEM, EDS, XRD as well their mechanical properties was determined through hardness measurements. Wear behavior and corrosion resistance were also evaluated.

## 2. Materials and Methods

### 2.1. Feedstock Powder

The Metco 439 NS powder (Oerlicon Metco^®^) with particle size distribution in the range of 11–63 µm was used for obtaining the coating deposition. The composite powder is manufactured by blending process. The feedstock material was characterized by scanning electron microscopy (SEM: Quanta FEG 250, FEI, Hillsboro, OR, USA) in order to study its morphology using backscattered electron detector (BSD) (same as Quanta FEG 250) and also elemental analysis was determined by energy dispersive X-ray spectroscopy (EDX with Apolo SSD: detector, EDAX Inc. Mahwah, NJ, USA). The chemical composition of the powder is presented in Table 1 (according to the manufacturer Oerlikon Metco^®^, Switzerland).

### 2.2. Laser Cladding

A coaxial laser cladding system was used to fabricate single and partially overlapped tracks of Metco 439 NS on low carbon steel base material. Different deposition speed was used as variable parameter. The experiments were carried out using a Coherent F1000 diode laser (Coherent, Santa Clara, CA, USA) equipped with a Precitec WC 50 cladding head and manipulated by a CLOOS 7 axes robot. An AT-1200HPHV Termach feeding system has delivered the powder to the laser head and as shielding and carrier gas Argon was used. The laser head was tilted at 3° in the cladding direction and a defocused laser beam of 2 mm was used for all the cladding tracks and coatings at a standoff distance of 12.5 mm. To optimize the processing speed, 10 single tracks of 90 mm were cladded by modifying the deposition speed by 10 cm/min starting from 15 cm/min up to 105 mm/min. All other parameters were kept constant. The cladding powder was kept at 720 W, powder feeding 4 g/min, preheating at 320 °C with Ar 14 L/min. The laser cladding parameters and the main geometrical dimension are summarized in Table 2.

The morphologies and microstructures of the cladded tracks and coatings were analyzed in cross-section by scanning electron microscopy (SEM: Quanta FEG 250, FEI, Hillsboro, OR, USA) using backscattered electron detector (BSD)and elemental analysis was performed by energy dispersive X-ray spectroscopy (EDX with Apolo SSD: detector, EDX with Apolo SSD: detector, EDAX Inc. Mahwah, NJ, USA). The dilution of cladded tracks was determined using an Olympus GX51 optical microscope (Olympus, Tokyo, Japan). The microhardness of the coatings was measured using a Future-Tech FM-700 microhardness tester (Future-tech Corp, Kanagawa, Japan). Ten HV01 indentations on the cross section of each coating were performed with the following set-up: load 100 gf and dwell time of 15 sec. The phases within the coating and feedstock powder were analyzed by mean of XRD technique using an Advanced D8 Discover Bruker diffractometer (Bruker, Billerica, MA, USA) with Kα1 = 1.5406 Å, 40 kW, 20 mA, a diffraction angle 2θ in the range of 20° to 100° and a scanning speed of 5°/min.

To evaluate the wear properties of the deposited coatings a ball-on-disk TR-20 tribometer was used (Ducom Instruments, Bangalore, India). Before testing, the samples were ground and polished until a mirror like surface was obtained. As counterpart a WC ball with a 6 mm diameter was used under an applied load of 15 N. The wear diameter was 12 mm, testing time 132 min and 2000 m sliding distance. Each test was repeated three time to obtain a good data repeatability. The wear rate was measured by using a 3D surface profilometer (Ducom Instruments, Bangalore, India). The morphologies of the worn surfaces were characterized using SEM and EDX techniques.

The corrosion behavior of the coating has been determined using a research grade SP-150 potentiostat/galvanostrat (Biologic, Seyssinet-Pariset, France) and a conventional three-electrodes electrochemical cell using the analyzed sample as working electrode. A 1 cm^2^ testing area was immersed in 3.5 wt.% NaCl solution. Each analysis was repeated three times in order to provide the reproducibility of the experiment.

## 3. Results and Discussions

### 3.1. Microstructural Characterization of the Feedstock Material

**Figure 1:**
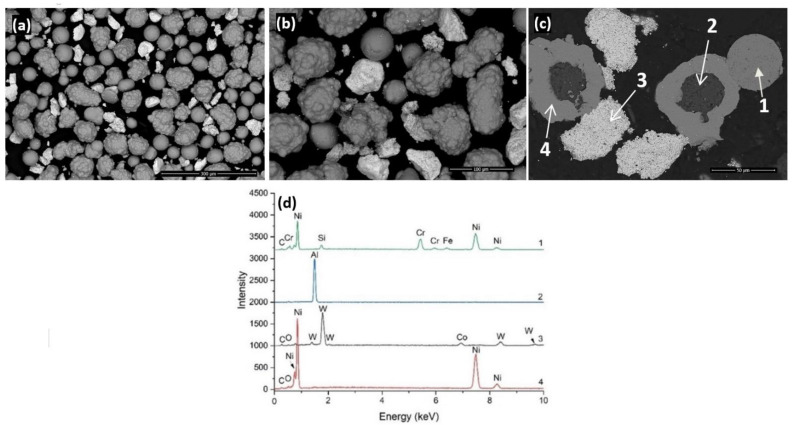
Back scattered electron micrograph of Metco 439 NS powder (**a**,**b**), powder cross-section (**c**) and EDX microanalysis (**d**).

As presented in Figure 1a and 1b the Metco 439 NS powder is composed from the Ni self-fluxing alloy and WC-Co powder with the addition of Ni-Al powder. The Ni-Al powder in combination with the self-fluxing alloy creates a self-fusing matrix. The mixture presents different morphologies. Spherical particles represent the self-fluxing alloy while the quasispherical aggregates represent the Ni-Al powder. The blocky morphology characterizes the WC-Co powder particles. This statement is confirmed by the powder particles cross-section presented in Figure 1b in accordance with the EDX spectra presented in Figure 1c. The Ni-Al powder is comprised of an Al core encapsulated in a Ni shell, enhancing the bonding between the coatings and the substrate.

### 3.2. Microstructural Characterization of Laser Cladded Single Tracks

**Figure 2:**
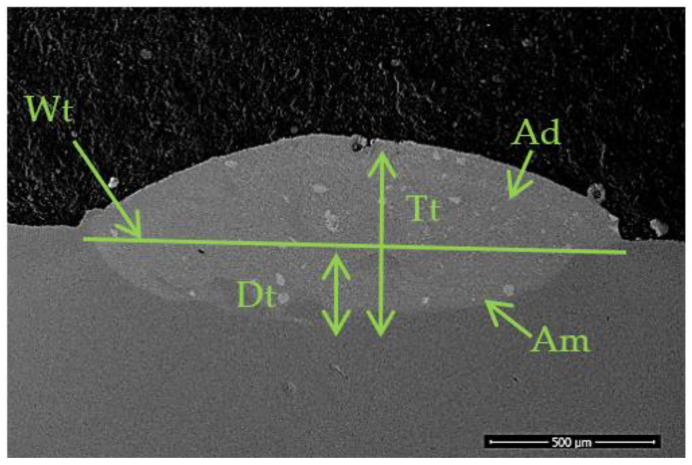
Back scattered electron micrograph of a laser cladded single track indicating the main geometrical dimensions.

In order to study the influence of cladding speed on the morphology of laser deposited single tracks the main geometrical dimensions were calculated for each track. For the measurements an optical microscope was used. Figure 2 presents a SEM micrograph on which the measured dimensions are presented. Thus, the indications are as follow: Wt—width of the laser track, Tt-–the total height, Dt-the depth of melted area, Ad—deposited, area and Am—melted area. Ad and Am were measured using the microscope’s software by selecting the contour of each area.

Dilution (η) can be defined as the ratio of the melted area in the substrate to the sum of the deposited area, above the substrate surface, and melted area (Equation (1)). Dilution determines the amount of liquid layer that forms on the substrate during cladding to ensure the bounding of the cladded coating to the substrate. If the values are too high, it means that elements from the substrate will mix in a high amount with the melting bath of the new deposited coating and might alter its properties.

(1) $$\eta =\frac{\mathrm{Am}}{\mathrm{Ad}+\mathrm{Am}}\times 100$$

The results of geometrical dimensions and the calculated dilution values are presented in Table 3.

From morphological point of view all tracks presented similar morphology with small differences regarding the geometrical dimensions. As seen from Table 3 the geometrical dimensions are strongly influenced by the cladding speed. Thus, while using a slower movement for the cladding laser head an increased amount of feedstock powder is melted, directly increasing the height and width of the cladding track. This also has a strong impact on the dilution within the deposited track. The slower the deposition speed the higher amount of dilution is observed. Using the same energy for melting the powder and substrate but varying the speed it is thought that the melted pool is keep somewhat longer under liquid form which has a considerable impact on the higher dilution rate. As the cladding speed increase, the dilution rate decreases but at a certain point. In our case until the value of 65 cm/min. After this value was reached, it can be seen that the dilution starts to increase again and the tracks are narrower and thinner. An explanation for this phenomenon might be that there is not enough time for the laser to melt the injected powder which is in lower amount due to the high speed thus the energy concentrates more on the substrate, resulting a melted pool rich in Fe. According to the data from Table 3, it might be concluded that the cladding speed used for the deposition of Track 6 led to the lowest degree of dilution. Among the parameters used to obtain the laser cladded single tracks, only five, the ones with the lowest dilution values, were selected as cladding parameters to deposit the WC-Co/NiCrBSi coatings.

### 3.3. Laser Cladding of WC-Co/NiCrBSi Coatings

In order to obtain the laser cladded coatings, the low carbon steel substrate was preheated at 320 °C to avoid cracking susceptibility. The coatings deposition was made using the five parameters which provided the lower dilution at an overlap degree of 45% with a focal point set at 0.75 mm. The selected cladding parameters function of speed and energy density are presented in Table 4.

### 3.4. Microstructural Characterization of Coatings

**Figure 3:**
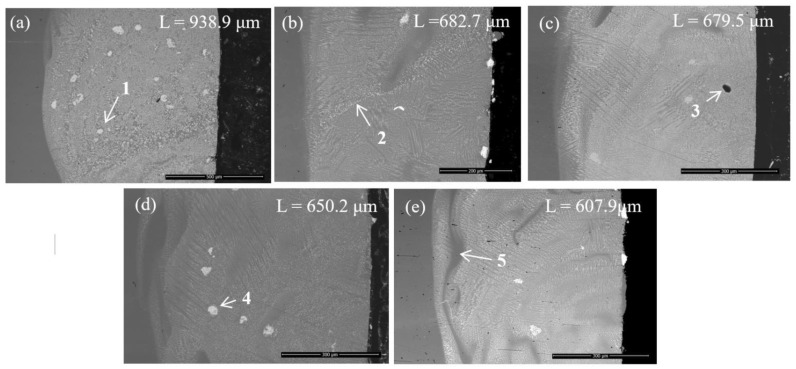
Back scattered electron micrographs of laser cladded WC-Co/NiCrBSi coatings indicating the morphology and thickness of coatings. Sample 1 (**a**), Sample 2 (**b**), Sample 3 (**c**), Sample 4 (**d**)and Sample 5 (**e**).

The effect of the cladding speed on Metco 439NS coatings was investigated using back scattered electrons. Figure 3 shows the cross-sectional view of samples deposited at different speeds on preheated substrate. Thick coatings with dense microstructure, good adherence to the substrate without visible macro-cracks with reduced porosity were obtained. It was noticed that there were no significant cracks across the coatings, indicating that the power and pre-heating played an important role as well during deposition. Thus, the thermal gradient and cooling rate within the molten pool managed to reduce the stress within the coatings and prevent macro-cracks formation. The thickness values were in the range of 938.9 µm and 607.9 µm. Figure 3 presents the morphology of laser cladded Metco 349 NS coatings manufactured by different cladding speeds at different magnifications in order to highlight the microstructure on the entire thickness of the coating. The coating thickness was measured using the SEM’s software. As it was expected, the coating thickness started to decrease while the cladding speed increased, in similar way as the single tracks. During laser cladding, the powder particles have different temperatures and velocities due to their different shape and chemical composition, thus they reach the substrate in molten, semi-molten and solid states. From the micrographs it can be noticed that an increased number of unmelted WC-Co powder particles are found in the lower area of the coatings. It is thought that the reason why the carbide-based powder particles tend to agglomerate in this area is the difference related to the higher density of carbide-based powder and the self-fluxing alloy. Due to a slower solidification rate of the Ni based self-fluxing alloy allows sufficient time to the carbide-based powder particles to immerse into the melted pool during spraying. Among the claddings, sample 1, 4 and 5 present a higher amount of unmelted WC-Co particles in the lower part of the coating. In Sample 1, due to a slower cladding speed, an increased amount of powder is deposited resulting a thicker coating which is favorable for the agglomeration of carbide-based particles. It might be noticed from the micrograph of Sample 1 presented in Figure 3 (label 1) that the carbide-based particles are not melted completely. The optimum cladding speed values are 55 and 65 cm/min, speeds that can provide a proper melting of WC-Co particles noticeable from the micrographs of Sample 2 and 3. Higher speeds than 65 cm/min led to thinner coatings obtained with reduced laser energy density. Thus, the carbide-based particles have not sufficient energy to melt as presented in Figure 3, Sample 4 (label 4). In some of the analyzed areas the interface between overlaid cladding tracks might be noticed (Sample 2–label 2) as well as some pores (Sample 3–label 3). In the micrographs of Sample 4 and 5 (label 5) darker areas might be observed especially in the lower part of the coatings. These are rich iron areas mixed with the coating due to the vortex produced during cladding by the laser beam.

**Figure 4:**
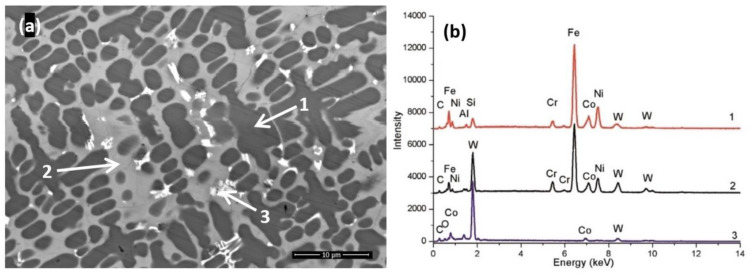
Back scattered electron micrograph of WC-Co/NiCrBSi coating (**a**) and EDX analysis (**b**).

The cladded layer is characterized (Figure 4) by a microstructure composed from fine dendrites with branch three growth and eutectic structure that is characteristic to Ni rich laser cladded layer, identified as well by S. Zhoua et al. [26]. The dendrites have high concentration of Ni solid solution with Cr, Fe, Co and Si (label 1) surrounded by interdendritic areas represented by a rich carbide content and Fe (label 2). It is thought that the diffusion of iron in the cladded coatings promotes the formation of coarse dendritic structures and influences their size and shape. The bright phases (label 3) in the micrograph, consist mainly of tungsten carbides rich regions, which are confirmed by the EDX spectra presented in Figure 4b. It can be seen that the WC particles have been dissolved and precipitated during solidification in different sizes and blocky shapes, randomly distributed throughout the coatings.

**Figure 5:**
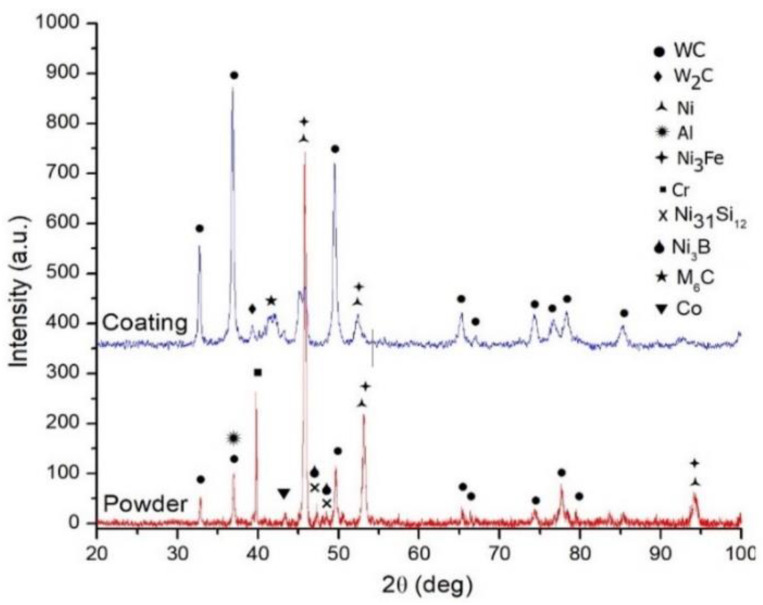
XRD patterns of the WC-Co/NiCrBSi coating and powder.

The phase composition of the feedstock powder and laser cladded coatings was evaluated by XRD. The coatings were polished prior analysis to reduce the contamination of the evaluated surface. Due to the fact that all the coatings have the same chemical composition and the EDX spectra were similar only the results for the power and one of the coatings were presented in Figure 5. The results showed that the feedstock powder is mainly composed from WC phase, γ-Ni, Cr, Al and Co. Ni_31_Si_12_ and Ni_3_B minor phases were identified, observed as well by [31]. Compared to the powder, the coatings present new phases which occurred during the cladding process due to phase transformation. The XRD analysis showed that the WC-Co/NiCrBSi coatings consists predominantly of the following constituents: WC, Ni, Ni_3_Fe along some minor phases like W_2_C and M_6_C (probably Co_3_W_3_C, Fe_3_W_3_C). The presence of W_2_C along with the presence of W within the matrix identified by EDX indicated that the WC-Co participated in the decarburization reactions during laser cladding. The excessive heating which occurs during laser cladding causes the WC particles at temperatures above 4200 °C to decompose resulting in the precipitation of W and C phases. It is likely that C reacts with other metals leading to the formation of carbides [32], e.g., M_6_C. The degree of carburization can be reduced during cladding but it cannot be completely eliminated. The high cooling rates experienced by the melted powder particles during cladding and the rapid solidification process of the molten bath are favorable to the formation of amorphous phases [33]. By preheating the substrate, the formation of amorphous phases was reduced considerably according to the XRD spectra from where it can be seen that the spectra display a widened diffraction peak at 43 °C θ indicating the presence of a small amount of amorphous phase within the coating as it was found as well by H. Guo et al. [32]. Other phases might as well be present but due to a very low diffraction peak, they were hard to be identified. Due to the lower amount of Al present into the Ni-Al powder particles and the presence of complex phases identified within the coatings, there were no peaks ascribed to Al.

### 3.5. Micro-Hardness and Fe Distribution

**Figure 6:**
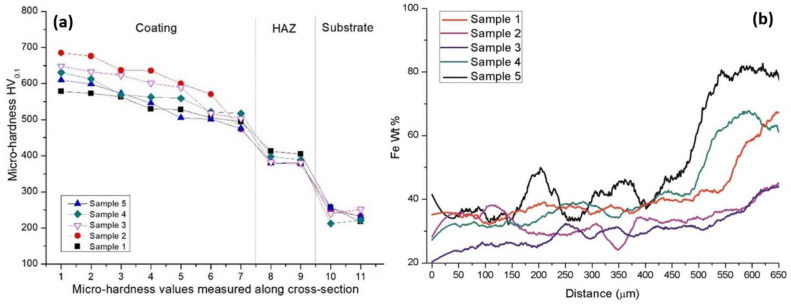
Microhardness measurements from top of the coating to the substrate (**a**) and Fe wt % content from top of the coatings to the substrate (**b**).

The micro-hardness was measured across the cross-section of the cladded coatings from the coating surface down to the heat affected zone (HAZ) and substrate. It can be observed from Figure 6a that the hardness of the coatings is much higher than the substrate and the values are particularly high in the upper part of the coatings. The fluctuations in hardness values are attributed to the carbide distribution throughout the coatings. The reason why the HAZ has higher values than the substrate is likely attributed to the metallurgical changes occurred due to rapid heating and solidification during laser cladding. The iron content was measured by EDX analysis across a line with start at the top part of the coating and ended up in the substrate as presented in Figure 6b. From the micro-hardness measurements and Fe distribution it might be observed that the cladding speed has a significant influence on the coatings. Among the coating, Sample 2 and 3 presented higher hardness values which are correlated to the lowest iron content found at the bottom of the coatings according to the analysis, meaning that this coatings in particularly have lower dilution. On the other hand, Sample 1, even if it was cladded with a reduced deposition speed, has lower hardness than Sample 2 and 3 which might be due to the formation of soft phases within the coating. Due to a lower speed, it is thought that the beam is focused somewhat longer on the molten bath which increases the compositional dilution having as result a higher content of soft phases within the coating. J. Amado at al. had similar results. In their research the lower deposition speed contributed directly to a higher energy density which led carbide dissolution and a higher level of dilution [34]. If the deposition speeds exceed 65 cm/min the beam has not enough time to melt the same amount of powder, thus a lower amount of powder is melted and much more energy is focused on the substrate increasing the iron content within the molten bath reducing in this way the hardness of the coatings. This is in accordance with the increased Fe content in samples 5 and 6 presented in Figure 6b. Similar behavior was observed by M. Vostřák et al. In their work the highest level of dilution was reached by using fastest cladding speed [35].

### 3.6. Tribological Properties in Dry Conditions

**Figure 7:**
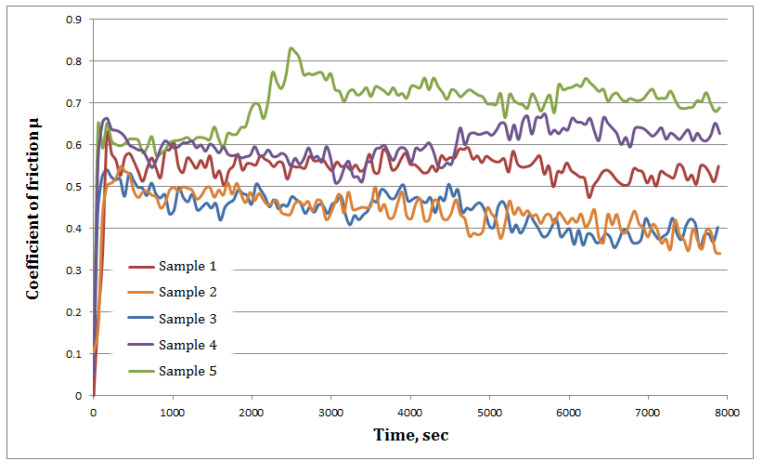
CoF evolution in time.

The coefficient of friction (Cof) as function of testing time for the cladded coatings are presented in Figure 7 and the resulted values are summarized in Table 5. At the beginning of the wear test, the Cof fluctuates because the contact between the coating and counter-body (the WC ball) is small with a large contact stress leading to an increased Cof. After that, the contact stress continues to decrease and the Cof tends to stabilize [36]. It can be also seen that all the coatings present uneven curves which is attributed to an uneven carbide distribution throughout the coatings. The smallest material loss was calculated for Samples 2 and 3 which had the lowest dilution and higher hardness values compared to the other coating.

The wear test results are shown in Table 5. It can be observed that the average wear index of Sample 2 and 3 is 0.0528 and 0.05 (mm^3^/N Km), respectively, are the lowest values among the tested samples. It was observed that the lower the iron content and the higher the hardness the lower the Cof, thus the wear loss. The possible reason for this might be the hard carbide particles embedded into a harder matrix with lower iron content which enhance the micro-cutting resistance which reduce the surface wear. Similar findings were reported by P. Zhang et al. [37].

**Figure 8:**
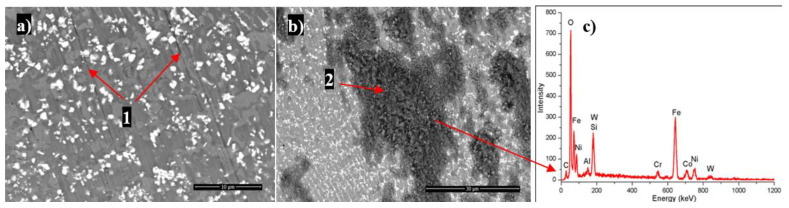
Back scattered electron micrograph of WC-Co/NiCrBSi wear scars after POD test (**a**); micro-ploughing–abrasive grooves (1), tribo-oxidation–migration of debris (**b**) along the surface (2), and EDX (**c**) spectra of debris agglomeration.

During ball-on-disk test micro-ploughing occurred (resulting in the formation of abrasive grooves which cut through the Ni rich matrix presented in Figure 8a, label 1) and triobo-oxidation through the formation of fine debris particles which were entrapped between the counter-body (the WC ball) and the surface of the cladded coatings acting as abrasive media (the dark areas on the surface presented in Figure 8b–label 2). The fine debris particles while entrapped between two surfaces were prone to oxidation according to the EDX spectrum presented in Figure 8c. It is thought that oxidation occurs during the wear test when the soft matrix debris reacts with the environment. Due to the presence of WC particles within the coatings, during the wear test this hard phase is pulled out from the softer matrix intensifying the wear mechanism. It can be concluded that the main wear mechanism is adhesive and abrasive wear. Similar conclusions were reported by Q. Bai et al. [36].

### 3.7. Corrosion Behavior of the Cladded Coatings

The corrosion behavior of the WC-Co/NiCrBSi samples was determined by potentiodynamic polarization studies. The evolution of the equilibrium potential was determined by measurement of the linear polarization, after the system had reached a quasi-stationary state (one hour).

**Figure 9:**
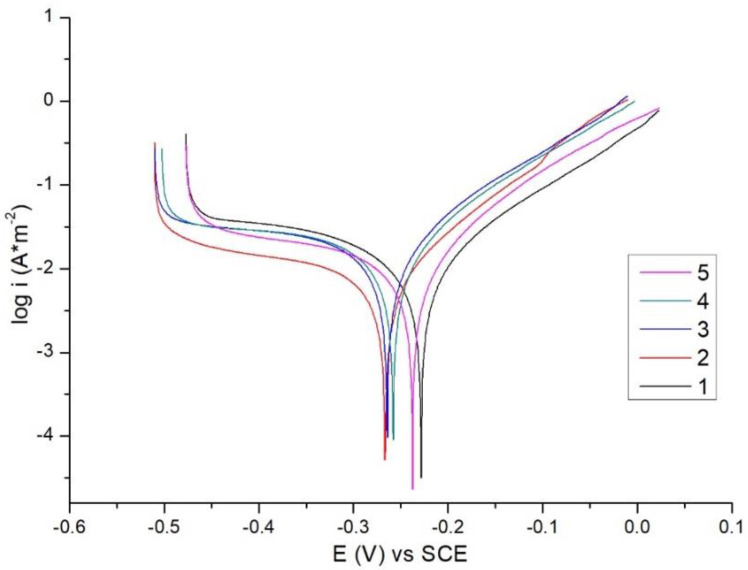
Potentiodynamic polarization curves for laser cladded NiCrBSi coatings in 3.5% NaCl solution at 22 °C.

Potentiodynamic polarization is used in order to determine the corrosion rate via the intensity of the corrosion current and Tafel slopes. A sweep potential of ±250 mV was applied against the open circuit potential and a scan speed of 1 mV/s was used. Thus, the corrosion rate can be calculated using the direct substitution of Tafel slope values. Linear polarization curves were recorded for WC-Co/NiCrBSi laser cladded coatings in 3.5% NaCl at 22 °C. Analyzing the polarization curves presented in Figure 9, samples 2, 3 and 4 showed a better corrosion resistance compared to the other investigated coatings. The plots shift towards lower passive current density and wide passive region due to dense coatings and lower dilution. These results are in tight connection with the presence of iron content within the coatings. In the present study, it can be concluded that if the iron content increases, thus the dilution increases leading to a reduced corrosion resistance.

In order to determine the afferent electrochemical parameters, the polarization curves were fitted, and the results are summarized in Table 6. Comparing the polarization curves and analyzing the electrochemical data it can be noticed that Sample 2 and 3 presented the lowest current density values which means the best corrosion resistance. These samples showed a dense structure and lower iron content within the coating which have a positive effect on the corrosion behavior of the coatings.

## 4. Conclusions

1. Using laser cladding and Metco 439 NS feedstock powder, dense coatings with reduced porosity without significant cracks might be manufactured.
2. The cladding speed has a major influence on the coating’s microstructure, wear resistance and corrosion behavior.
3. Dense and crack free coatings with a desirable distribution of carbides can be produced by optimization of the laser cladding speed. Moreover, hardness increasing was obtained by reducing the dilution between the coating and substrate.
4. Due to high temperatures occurred during laser cladding, new phases have formed according to the XRD analysis like Ni_3_Fe along with M_6_C and W_2_C minor phases; by preheating the substrate the formation of amorphous phases was reduced considerably.
5. Optimizing the cladding speed proved to increase the wear resistance and enhance the corrosion behavior by controlling the iron content within the coatings.

## Figures and Tables

**Table 1:** Chemical composition of Metco 439 NS powder.

Chemical Composition	WC-12Co	Ni	Cr	Al	Fe	Si	B	C
Element [%]	50	balance	5.8	2.8	1.4	1.1	1.3	0.3

**Table 2:** Cladding parameters for individual tracks.

Parameters Track No.	Power [W]	Cladding Speed [cm/min]	Powder Feeding [g/min]	T Preheat [°C]	Ar [L/min]
Track 1	720	15	4	320	14
Track 2		25			
Track 3		35			
Track 4		45			
Track 5		55			
Track 6		65			
Track 7		75			
Track 8		85			
Track 9		95			
Track 10		105			

**Table 3:** Cladding track dimensions and dilution calculus.

Dimensions and Dilution Track	Wt [mm]	Dt [µm]	Tt [µm]	Am [µm^2^]	Ac [µm^2^]	η%
Track 1	2.257	399.1	781.6	794,251.4	615,719.36	56.33
Track 2	2.268	393.6	785.8	757,069.93	597,623.11	55.88
Track 3	2.217	386.2	779.3	690,433.92	613,919.49	52.93
Track 4	2.207	381.3	771.6	653,186.24	606,391.36	51.85
Track 5	2.308	295	760.8	417,360.39	461,920.95	47.46
Track 6	2.229	343.5	606.1	492,017.64	801,300.36	38.04
Track 7	1.801	311.6	661	452,454.7	590,101.93	43.39
Track 8	1.633	265.8	563.9	466,151.94	432,516.01	51.87
Track 9	1.777	224.1	349.3	174,467.22	155,222.13	52.91
Track 10	1.608	259.4	469.4	194,068.92	162,457.3	54.43

**Table 4:** Laser cladding parameters.

Parameter Coating	Power [W]	Cladding Speed [cm/min]	Energy Density [J/mm^2^]	Powder Feeding [g/min]	T Preheat [°C]	Ar [L/min]
Coating 1	720	45	80	4	320	14
Coating 2		55	65.4			
Coating 3		65	55.3			
Coating 4		75	48			
Coating 5		85	42.3			

**Table 5:** Sliding wear test results.

Parameter Sample	μ	m_i_ [g]	m_f_ [g]	Δm [mg]	Wear Index [mm^3^/N Km]
Sample 1	0.557	34.689	34.687	1.46	0.125
Sample 2	0.543	39.671	39.67	1.61	0.0528
Sample 3	0.438	35.174	35.172	1.91	0.05
Sample 4	0.695	34.38	34.378	1.97	0.721
Sample 5	0.600	35.405	35.403	2.65	0.735

**Table 6:** Corrosion test results.

Sample	E [mV]	I_corr_ [µA/cm^2^]	Corr. Rate [mm/Year]
1	−239.202	11.569	0.28
2	−231.307	11.071	0.26
3	−266.21	8.056	0.18
4	−263.471	19.067	0.44
5	−257.958	17.771	0.41

## Data Availability

The data presented in this study are available on request from the corresponding author.
